# Platelet factor-4 and its p17-70 peptide inhibit myeloma proliferation and angiogenesis in vivo

**DOI:** 10.1186/1471-2407-11-261

**Published:** 2011-06-21

**Authors:** Longjiang Yang, Juan Du, Jian Hou, Hua Jiang, Jianfeng Zou

**Affiliations:** 1 Department of Hematology, Changzheng Hospital, The Second Military Medical University, 415 Fengyang Rd, Shanghai 200003, China

## Abstract

**Background:**

Angiogenesis plays an important role in the development of multiple myeloma (MM). The interaction between MM cells and the bone marrow microenvironment stimulates the proliferation and migration of endothelial progenitor cells (EPCs). Vascular endothelial growth factor (VEGF) contributes to the formation of new blood vessels by actively recruiting circulating EPCs. The production of proangiogenic and antiangiogenic factors is also dysregulated in MM. Platelet factor 4 (PF4) is a potent angiostatic cytokine that inhibits angiogenesis and tumor growth in several animal models.

**Methods:**

In this study, we stably transfected human myeloma cell lines with the PF4 gene or the sequence encoding its more potent p17-70 peptide and investigated the effects of PF4 and p17-70 on angiogenesis and tumor growth *in vitro *and in a SCID-rab myeloma model.

**Results:**

PF4 and p17-70 significantly attenuated VEGF production, both *in vitro *and *in vivo*. In a migration study using a Transwell system, PF4 or p17-70 markedly suppressed the migration of co-cultured human endothelial progenitor cells. PF4 or p17-70 also caused a significant reduction in microvessel densities in myeloma xenografts and markedly reduced the tumor volume in the SCID mice. Kaplan-Meier analysis demonstrated that PF4 and p17-70 significantly extended the overall survival of SCID mice bearing human myeloma xenografts.

**Conclusions:**

Our findings indicate that PF4 or p17-70 could be valuable in combating multiple myeloma by disrupting tumor angiogenesis.

## Background

Multiple myeloma (MM) is a plasma cell neoplasm characterized by skeletal destruction, renal failure, anemia, and hypercalcemia, and is the second most common hematological neoplasm after lymphoma [1,2]. Novel agents such as the proteasome inhibitor bortezomib, thalidomide, and lenalidomide have so far only produced a modest improvement in the therapeutic outcomes of MM patients [3] and it remains an incurable disease.

The therapeutic effect of thalidomide is partly attributed to its antiangiogenic action on MM cells [4]. Bone marrow angiogenesis occurs in MM patients and correlates with the treatment response and survival [5]. Angiogenesis involves the development of blood vessels of capillary origin, a process tightly controlled by proangiogenic factors such as fibroblast growth factor 2 (FGF2) and vascular endothelial growth factor (VEGF). Human endothelial progenitor cells (EPCs) contribute to this neovascularization [6], and consequently, the inhibition of EPC recruitment can lead to the suppression of tumor angiogenesis and hence the inhibition of tumor growth, offering a promising therapeutic target [7,8].

The chemokine platelet factor 4 (PF4) is a 70-residue polypeptide with pleiotropic biological effects. It is released from platelet α-granules during platelet aggregation [9,10]. As well as being synthesized in platelets or megakaryocytes, is also produced in other cell types, including monocytes, T-cells, and neutrophils [11]. PF4 exerts potent angiostatic effects by inhibiting endothelial cell proliferation, and this effect is localized to the amino acid residues 17-70 of the molecule [12,13]. PF4 and p17-70 suppress angiogenesis by inhibiting proangiogenic factors such as FGF2 [14-17]. PF4 also inhibits the function of VEGF by disrupting the binding of VEGF to its receptor [18] and suppressing the VEGF-induced intracellular signaling cascade [19].

Aberrant angiogenesis arising from the dysregulation of the production of proangiogenic and antiangiogenic factors is observed in MM patients. Our genomic analysis of human myeloma cells showed that the PF4 gene is frequently silenced by promoter hypermethylation, which could contribute to the aberrant angiogenesis in MM [20]. Therefore, we hypothesized that the overexpression of the PF4 gene or p17-70 in MM cells would inhibit the growth of myeloma by inhibiting the functions of proangiogenic factors such as FGF2 and VEGF. In this study, we investigated the effects of the overexpression of the PF4 gene or p17-70 in MM cells on VEGF production and also examined its effects on angiogenesis in cell lines and a mouse xenograft model.

## Methods

### Cell lines

The myeloma-derived cell lines U266, RPMI8226, and LP-1 were purchased from the American Type Culture Collection (Manassas, VA) and cultured in RPMI-1640 medium containing 10% fetal bovine serum (FBS), penicillin (10 IU/ml) and streptomycin (100 μg/ml) (Invitrogen, Carlsbad, CA) at 37°C with 5% CO_2_. HEK 293T cells were also grown in Dulbecco's modified Eagle's medium (Invitrogen) supplemented with 10% FBS and 1% penicillin/streptomycin under the same conditions. Human EPCs were cultured and maintained as previously described [21].

### Lentiviral Transfection

The entire open reading frame of *PF4 *(NCBI reference sequence: NM_002619) was amplified using the PF4 primers: forward 5"-GCGAATTCGCCACCATGGAAGCGGAAGAAGATGGGG-3" and reverse 5"-CCGCTCGAGTTAACTCTCCAAAAGTTTC-3". The sequence encoding p17-70 was amplified with primers: forward 5"-GCGAATTCGCCACCATGACCTCCCAGGTCCGTCCCAG-3" and reverse 5'- CCGCTCGAGTTAACTCTCCAAAAGTTTC-3". The PCR was performed in a 50 μl reaction containing 5 μl of 10 × Ex buffer, 2.5 mM dNTPs, 10 μL of each primer, 0.25 μL of Ex *Taq *DNA polymerase (Takara, Millipore), and 1 μl of DNA template. To amplify the PF4 gene or p17-70, the PCR was run for 35 cycles of 94°C for 1 min, at 60°C for 1 min, and at 72°C for 1 min, followed by a final extension at 72°C for 10 min. The PCR products were gel purified and the PF4 or p17-70 cDNA was inserted into the *Eco*RI/*Xho*I sites of the vector pIRES2-ZsGreen1 (BD Biosciences) to generate two new vectors, pPF4-IRES2-ZsGreen1 and psPF4-IRES2-ZsGreen1. The two plasmids were confirmed by sequencing (Sangon, Shanghai, China). We next transfected HEK 293T cells with the expression vectors pPF4-IRES2-ZsGreen1 or psPF4-IRES2-ZsGreen1 and the packaging plasmid mix, as instructed by the manufacturer (Clontech Laboratories, Mountain View, CA). After incubation for 36-72 hours, the lentiviral vectors were harvested from the supernatants using the QuickTiter™ Lentivirus Concentration and Purification Kit (Cell Biolabs, San Diego, CA). The lentiviral transfections were performed at a minimum effective multiplicity of infection of 20. The efficiency of transfection was estimated by counting the percentage of green fluorescent protein (GFP)-positive cells 48 h after transfection. The transfected cell lines U266, RPMI8226, and LP-1 were treated with G418 (Sigma, St Louis, MO) at increasing concentrations from 200 to 1,000 μg/mL for 6 days, and the stable cell lines were then screened using the limited dilution method, as described previously [22].

### Immunoblotting studies and enzyme-linked immunosorbent assay (ELISA)

The production of PF4 and p17-70 in the U266, RPMI8226, and LP-1 cell lines, and the appropriate controls, was examined by immunoblotting studies according to a previous report [23,24], The antibodies used were anti-PF4 antibody (Cell Signaling, Danvers, MA) and anti-β-actin antibody (Santa Cruz Biotechnology, Santa Cruz, CA). The protein contents were determined with the Bradford method and equal amounts of cellular lysates were resolved by gradient (4-12%) sodium dodecyl sulfate-polyacrylamide gel electrophoresis (SDS-PAGE; Bio-Rad, Hercules, CA). The VEGF levels in the supernatants of the cell lines and controls were measured with a Quantikine VEGF ELISA Kit (R&D Systems, Minneapolis, MN), according to the manufacturer's protocol.

### Coculture of MM cells with human EPCs

Mononuclear cells were isolated from human EDTA-blood by centrifugation on Ficoll-Paque Plus gradient (Amersham Biosciences). The isolated cells were suspended in Medium 199 with 20% New-Born Calf Serum (NBCS), 30 ng/ml recombinated human vascular endothelial growth factor (rh-VEGF) and 6 ng/ml recombinated human basic fibroblast growth factor (rh-b-FGF), as previously described [25]. Human EPCs were washed three times with phosphate-buffered saline to remove any loosely attached cells. The adherent cells (1 × 10^5 ^cells) were then harvested after synchronization for 24 h with serum-free medium and cocultured with the appropriate myeloma cell lines (1 × 10^5 ^cells) in RPMI-1640 medium containing 10% FBS in a Transwell system (8 μm, Costar, Cambridge, MA). Migration assay was performed as previous description [26]. Cells were dispersed onto filters, and were then challenged by the addition of 600 μl of a chemoattractant solution composed of conditioned media, and 50 ng/ml rh-VEGF and 10 ng/ml rh-b-FGF to the lower compartments. Migration was allowed to proceed for 24 h at 37°C under hypoxia. Cells remaining attached to the upper surface of the filters were carefully removed with cotton swabs. Cells that had migrated to the lower surface of the filters were fixed with 10% neutral buffered formalin, stained with 0.1% crystal violet/20% MeOH, and counted. The average number of migrating cells per field was assessed by counting at least four random fields per filter.

### Proliferations of human EPCs

The EPC proliferation was determined by colorimetric 3-(4, 5-dimethylthiazol-2-yl)-2,5 -diphenyltetrazollium bromide (MTT) assay. MTT (Sigma) was dissolved in phosphate-buffered saline (PBS) at 5 mg/ml and used to measure cell proliferation. Five thousand colony-derived cells were cultured in 96-well culture plate (200 μl/well). The cells were resuspended in RPMI-1640 containing 10% FBS medium were divided into groups according to the study design and were cultured for 0, 4, 8,12, and 24 hours. And then serum-free medium (180 μl) with MTT (20 μl, 5 mg/ml) was added and incubated for another 4 h. The supernatant was discarded by aspiration and EPCs preparation was shaken with 150 μL DMSO for 10 min prior to the measurement of OD value at 490 nm on a micro oscillating device.

### Terminal-deoxynucleotidyl-transferase-mediated biotin-dUTP nick end labeling (TUNEL) and annexin V/propidium iodide staining

Human EPCs were collected, applied to prepared cytospin slides (10^5 ^cells/slide), and fixed in 10% phosphate-buffered formalin for 20 min in freshly prepared 4% paraformaldehyde. The TUNEL assay was performed using the Klenow FragEL™ DNA Fragmentation Detection Kit (Calbiochem, Darmstadt, Germany) according to the manufacturer's instructions. The EPCs cocultured with myeloma cells for 48 h were collected and stained with an annexin V-propidium iodide (PI) detection kit for apoptosis (Caltag Laboratories, Burlingame, CA), as instructed by the manufacturer, and analyzed by flow cytometry on a FACScan flow cytometer (Becton Dickinson, San Jose, CA). The results are expressed as the percentage of apoptotic cells that stained positively for annexin V. The adherent EPCs were also stained for annexin V-propidium iodide and the total number of apoptotic cells was counted under a Zeiss AX10 Observer A1 microscope (Delta Optical Instruments, Thornwood, NY).

### SCID-rab myeloma model

SCID mice, 6-8 weeks old and weighing 18-20 g, were used to establish the SCID-rab myeloma model [27]. Four-week-old rabbits were anesthetized with phenobarbital sodium and killed by cervical dislocation. Their tibiae were removed and cut into bone blocks of 5 × 5 × 10 mm^3^, which were then inserted subcutaneously through a 5 mm incision onto the ventral surfaces of the SCID mice. Four weeks was allowed for the engraftment of the bones to occur. For each mouse, 3-5 × 10^6 ^U266, RPMI8226, or LP-1 cells stably transfected with the PF4 or p17-70 cDNA or the control plasmid were injected directly into the implanted rabbit tibiae. Eight SCID mice were injected with each of the appropriate tumor cells. The mice were bled from the tail vein every 2 weeks and changes in the levels of circulating human immunoglobulin light chain proteins and VEGF were measured by ELISA. The tumor volume was calculated with the following formula: volume = (4/3) × 3.14 × (long diameter/2) (short diameter/2) (long diameter + short diameter)/4. The growth of the tumor xenografts in the SCID mice was examined by normal and X-ray photography 12 weeks after the inoculation of the tumor cells. After the animals were killed, the gross appearance of the tumors was examined and tissue sections were prepared from the tibiae bearing human myeloma xenografts for hematoxylin-eosin staining and for immunohistochemical staining with antibody directed against CD31 (Santa Cruz Biotechnology). The microvessel density (MVD) within the tumor was determined by immunohistochemistry for CD31.

### Statistical analysis

Each experiment was performed independently at least three times. The data were plotted as mean ± SD and analyzed for statistical significance between groups with the use of ANOVA followed by post hoc Student-Newman-Keuls test. A two-tailed paired Student's t test was used to compare significant difference between groups in human immunoglobulin light chain protein levels and human VEGF levels. For the in vivo study, the data were plotted as mean ± SD and analyzed for statistical significance with the use of repeated measures ANOVA. A *P *value of less than 0.05 was considered statistically significant. The survival rates were compared using the Kaplan-Meier method and the log-rank test.

## Results

### PF4 and p17-70 attenuate VEGF production in myeloma cells *in vitro*

**Figure 1:**
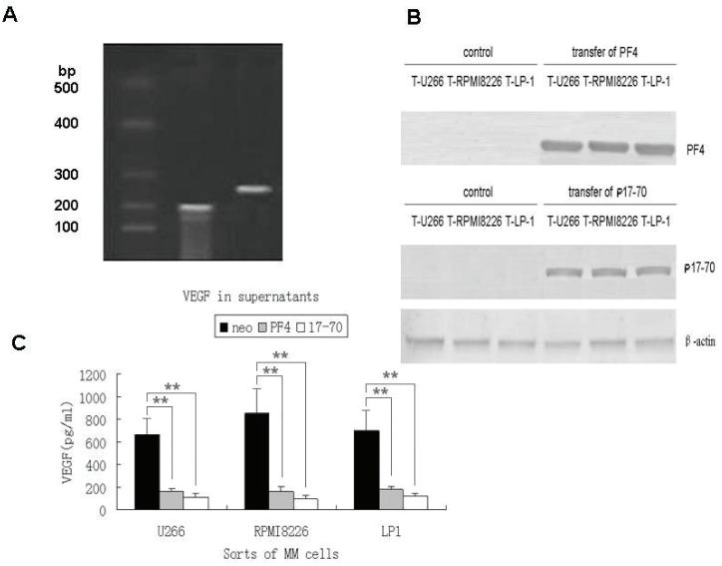
**PF4 and p17-70 attenuate VEGF production in myeloma cells *in vitro***. (A) Identification of the expression and integration of foreign genes (A: 1-2): RT-PCR analysis of RNA in U266 cells transfected with different retroviruses. M: DNA size marker; lane 1: p17-70-infected U266 cells (150 bp); lane 2: PF4-infected U266 cells (170 bp). (B) Western blotting assay of selected MM cells. Control groups: U266-neo, RPMI8226-neo, and LP-1-neo; p17-70 groups: U266-p17-70, RPMI8226-p17-70, and LP-1-p17-70. (C) VEGF levels in the supernatants of three sorts of MM cells. There were significant differences in the VEGF levels in the supernatants of the U266 cells among the groups (PF4: 160.36 ± 8.80 pg/ml, p17-70: 98.25 ± 9.65 pg/ml, U266-neo: 663.58 ± 142.16 pg/ml, both of *P *< 0.01). There were significant differences in the VEGF levels in the supernatants of the RPMI8226 cells among the groups (PF4: 147.61 ± 5.15 pg/ml, p17-70: 86.73 ± 4.81 pg/ml, RPMI8226-neo: 848.61 ± 216.15 pg/ml both of *P *< 0.01). There were significant differences in the VEGF levels in the supernatants of the LP-1 cells among the groups (PF4: 167.92 ± 10.28 pg/ml, p17-70: 109.32 ± 5.28 pg/ml pg/ml, LP-1-neo: 701.73 ± 192.81 pg/ml both of *P *< 0.01). Figures represents the three independent experiments done in triplicate (**, *P *< 0.01).

We generated stable human myeloma cell lines (U266, RPMI8226, and LP-1) expressing PF4 or p17-70. RT-PCR analysis revealed that the stable U266 cell line expressed the appropriately sized PF4 or p17-70 products (Figure 1A). The production of the U266, RPMI8226, and LP-1 cells showed that PF4 or p17-70 was effectively produced from these cell lines transfected with either the PF4 gene or sequence encoding p17-70, whereas neither of them was detected in the control cells in western blotting assay (Figure 1B). In an analysis of the effects of PF4 and p17-70 on the expression of VEGF, we found that the levels of VEGF in the supernatants of the U266, RPMI8226, and LP-1 cells stably expressing the PF4 gene were significant reduced to 160.36 ± 8.80 pg/ml, 147.61 ± 5.15 pg/ml, and 167.92 ± 10.28 pg/ml, respectively, compared with their control counterparts U266-neo 663.58 ± 142.16 pg/ml, RPMI8226-neo 848.61 ± 216.15 pg/ml, and LP-1-neo 701.73 ± 192.81 pg/ml (all *P *< 0.01; Figure 1C). Similar results were observed for p17-70, and the levels of VEGF in the supernatants of the U266, RPMI8226, and LP-1 cells stably expressing the p17-70 gene fragment were apparently reduced to 98.25 ± 9.65 pg/ml, 86.73 ± 4.81 pg/ml, and 109.32 ± 5.28 pg/ml, respectively (all *P *< 0.01).

### Activation in human EPCs by PF4 or p17-70

**Figure 2:**
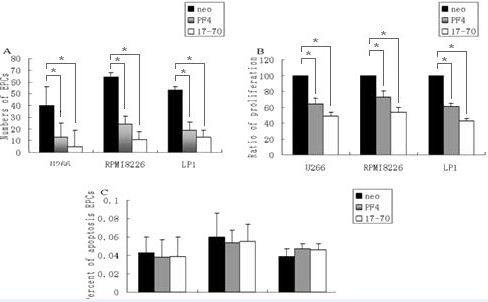
**The EPCs properties in three sorts of MM cells**. (A) The migration capacities of EPCs detected by transwell migration (*P *= 0.024). (B) The proliferation capacities of EPCs determined by MTT assay (*P *= 0.019). (C) The apoptosis capacities of EPCs used annexin V/PI staining assay (*P *= 0.34). Figures represents the three independent experiments done in triplicate (*, *P *< 0.05).

We examined the effects of PF4 and p17-70 on the migratory capacity of activated human EPCs. EPCs cocultured with U266, RPMI8226, or LP-1 myeloma cells stably expressing PF4 were significantly reduced to 70%, 61%, and 67%, respectively, whereas those expressing the p17-70 fragment were reduced to 87%, 85% and 77%, respectively, compared with the control cells (all *P *< 0.05; Figure 2A). We further investigated the effects of PF4 and the p17-70 fragment on cell proliferation, and showed that both of them expressed in U266, RPMI8226, and LP-1 myeloma cells markedly inhibited the proliferation of human EPCs to 67%, 73%, 58%, and 49%, 56%, 42%, respectively (all *P *< 0.05; Figure 2B). However, no significant difference was observed in the apoptosis of the cell lines and the control cells (all *P *> 0.05; Figure 2C).

### PF4 and p17-70 attenuated the growth of myeloma in SCID-rab mice

**Figure 3:**
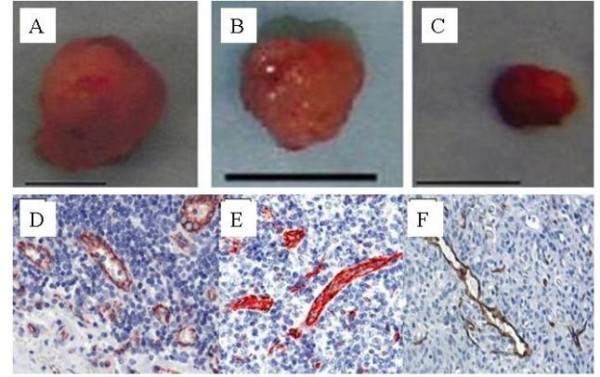
**Photographs of representative tumors and immunostaining of representative tumors**. Photographs of representative tumors from the U266-neo (151.3 ± 14.5 mm^3^) (A), U266-PF4 (102.3 ± 19.8 mm^3^) (B), and U266-p17-70 (76.6 ± 7.9 mm^3^) groups (C). Immunostained tumor tissue sections from representative tumors from the U266-neo (D), U266-PF4 (E), and U266-p17-70 groups (F) are shown. Micrographs were taken at a magnification of 400×, except the section from a U266-p17-70 mouse, which was magnified 200 ×, which was processed by GIMP2 software.

We established a SCID-rab model of myeloma. Figure 3 shows in photographs and X-ray photographs that substantial tumors developed after the 12 weeks after the SCID-rab mice were inoculated with U266 cells. Similar results were observed in the SCID-rab mice inoculated with RPMI8226 or LP-1 cells (data not shown). The control mice had tumor sizes of 151.3 ± 14.5 mm^3 ^(Figure 3A), 163.5 ± 16.4 mm^3^, and 200.3 ± 16.7 mm^3 ^after inoculation with U266, RPMI8226, and LP-1, respectively. In contrast, the tumor sizes in the SCID-rab mice inoculated with myeloma cells stably expressing the PF4 gene was significantly smaller (102.3 ± 19.8 mm^3^, 118.2 ± 17.9 mm^3^, and 144.9 ± 15.6 mm^3 ^for U266 [Figure 3B], RPMI8226, and LP-1, respectively; *P *< 0.05) and in those inoculated with myeloma cells stably expressing the p17-70 fragment (76.6 ± 7.9 mm^3^, 85.4 ± 8.2 mm^3^, and 87.1 ± 9.6 mm^3 ^for U266 [Figure 3C], RPMI8226, and LP-1 cells, respectively; *P *< 0.05), suggesting that PF4 and p17-70 attenuated the growth of the myelomas in the SCID-rab mice.

### PF4 and p17-70 suppressed angiogenesis of myelomas in SCID-rab mice

We further assessed the MVD in tumor sections stained with anti-CD31 antibody. The blood vessel densities of the tumors from the U266-neo (30 ± 3 vessels/field) (Figure 3D), which was higher than U266-PF4 (15 ± 2 vessels/field) (*P *= 0.036, Figure 3E). The RPMI8226-neo (36 ± 4 vessels/field) was higher than RPMI8226-PF4 (17 ± 3 vessels/field) (*P *= 0.039), while LP-1-neo (28 ± 3 vessels/field) groups were higher thanthe blood vessel densities of the LP-1-PF4 (16 ± 3 vessels/field) groups too (*P *= 0.041). The similar results were observed among p17-70 treated with U266 (Figure 3F), RPMI8226, and LP-1 myeloma cells when compared with control group (*P *< 0.05 in all three groups, data not shown).

### PF4 and p17-70 attenuate human immunoglobulin light chain protein levels in myeloma cells of SCID-rab mice

**Figure 4:**
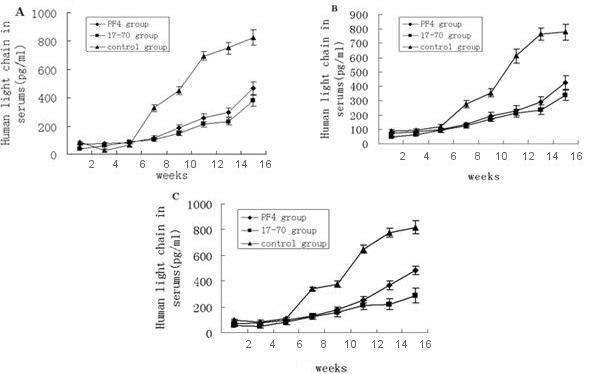
**Human immunoglobulin light chain protein levels in the sera of mice after treated with three sorts of MM cells**. There were significant differences in the human immunoglobulin light chain protein levels in the sera of the mice in the three groups treated with three sorts of MM cells (all *P *< 0.01). (A) Light chain levels of SCID-rab MM mice with tumors derived from U266 cells at 16 weeks [PF4 (346.3 ± 45.4 pg/ml); p17-70 (327.8 ± 34.2 pg/ml); U266-neo (823.2 ± 53.7 pg/ml)]. (B) Light chain levels of SCID-rab MM mice with tumors derived from RPMI8226 cells at 16 weeks [RPMI8226: PF4 (325.6 ± 47.3 pg/ml); p17-70 gene fragment (338.0 ± 36.1 pg/ml); RPMI8226-neo (778.7 ± 55.1)]. (C) Light chain levels of SCID-rab MM mice with tumors derived from LP-1 cells at 16 weeks [PF4 (334.6 ± 53.1 pg/ml); p17-70 (289.0 ± 32.7 pg/ml); LP-1-neo (819.7 ± 56.3 pg/ml)].

We then determined whether the reduced tumor size in the SCID-rab mice inoculated with myeloma cells stably expressing PF4 or p17-70 was associated with a reduction in the production of the human immunoglobulin light chain. The serum levels of human immunoglobulin light chain in these SCID-rab mice were significantly reduced compared with those in the control cells. In U266 cells, the serum levels of human immunoglobulin light chain protein levels in the SCID mice bearing human myeloma xenografts stably expressing PF4 (346.3 ± 45.4 pg/ml) or the p17-70 gene fragment (327.8 ± 34.2 pg/ml) compared to control (823.2 ± 53.7 pg/ml) from U266 cells at 16 weeks, that the experiments groups in RPMI8226 and LP-1 cells were observed the similar results [RPMI8226: PF4 (325.6 ± 47.3 pg/ml); p17-70 (338.0 ± 36.1 pg/ml); RPMI8226-neo (778.7 ± 55.1), LP-1: PF4 (334.6 ± 53.1 pg/ml); p17-70 (289.0 ± 32.7 pg/ml); LP-1-neo (819.7 ± 56.3 pg/ml)] (*P *< 0.01 in all three groups; Figure 4).

### PF4 and p17-70 inhibited VEGF production of myeloma in SCID-rab mice

**Figure 5:**
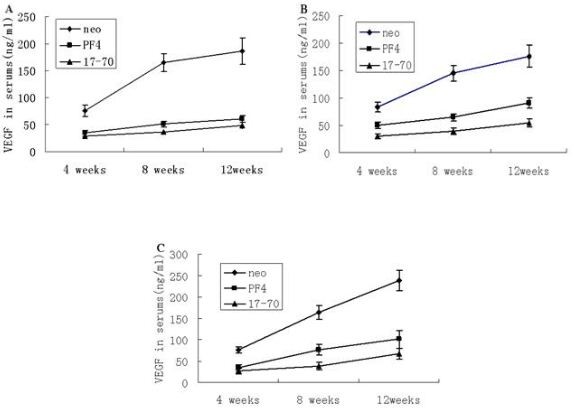
**Human VEGF levels in the sera of mice**. (A) SCID-rab MM mice with tumors derived from U266 cells. There were significant differences in VEGF levels in the sera of mice among the groups [PF4 (191.1 ± 26.5 pg/ml); p17-70 (183.7 ± 12.6 pg/ml); U266-neo (468.1 ± 42.8 pg/ml), *P *= 0.002]. (B) SCID-rab MM mice with tumors derived from RPMI8226 cells. There were significant differences in VEGF levels in the sera of mice among the groups [PF4 (155.3 ± 16.5 pg/ml); p17-70 (130.5 ± 18.3 pg/ml); RPMI8226-neo (410.7 ± 30.8 pg/ml), *P *= 0.001]. (C) SCID-rab MM mice with tumors derived from LP-1 cells. There were significant differences in VEGF levels in the sera of mice among the groups [PF4 (155.3 ± 16.5 pg/ml); p17-70 (196.3 ± 20.7 pg/ml); LP-1-neo (436.3 ± 34.5 pg/ml)*, P *= 0.001]

Because local blood vessel formation is associated with the growth and metastasis of tumors, we investigated whether PF4 and p17-70 inhibited the production of VEGF in SCID mice bearing human myeloma xenografts. We found that PF4 or p17-70 caused a significant reduction in the serum levels of VEGF in the SCID mice bearing human myeloma xenografts stably expressing PF4 (191.1 ± 26.5 pg/ml) or the p17-70 gene fragment (183.7 ± 12.6 pg/ml) compared to control (468.1 ± 42.8 pg/ml) from U266 cells at 12 weeks, that the experiments groups in RPMI8226 and LP-1 cells were observed the similar results, which were the serum levels of VEGF in RPMI8226 expressing PF4 (155.3 ± 16.5 pg/ml), the p17-70 gene fragment (130.5 ± 18.3 pg/ml) compared to control (410.7 ± 30.8 pg/ml), and in LP-1 expressing PF4 (155.3 ± 16.5 pg/ml), the p17-70 gene fragment (196.3 ± 20.7 pg/ml) compared to control (436.3 ± 34.5 pg/ml) (*P *< 0.01 in all three groups; Figure 5).

### PF4 and p17-70 extended the survival of SCID mice bearing human myeloma xenografts

**Figure 6:**
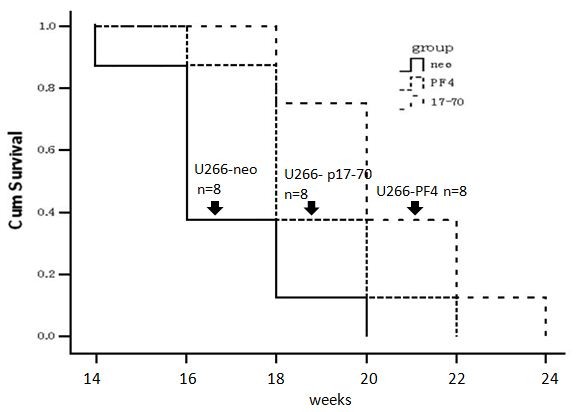
**Kaplan-Meier plot of OS in SCID-rab mice with tumors derived from U266 cells**. The median OS in U266-PF4 and p17-70-U266 were 18 weeks and 20 weeks, respectively (*P *= 0.004).

We also examined the effects of PF4 and p17-70 on the overall survival (OS) of SCID mice bearing human U266 myeloma xenografts. All the SCID mice survived longer than 14 weeks, whereas only one SCID mouse from the control group was dead at 14 weeks. Most importantly, animals bearing a U266 xenograft stably expressing PF4 or the p17-70 fragment (median OS of 18 or 20 weeks, respectively) survived significantly longer than the control animals (*P *= 0.004; Figure 6), further highlighting the potential of PF4 or the p17-70 fragment expression effects. The similar results were observed of SCID mice bearing human RPMI8226 or LP-1 myeloma xenografts (data not shown).

## Discussion

Tumors recruit neighboring blood vessels and vascular endothelial cells to support their own malignant growth. In the process, tumor cells secrete trophic angiogenic molecules, such as VEGF, that induce the proliferation and migration of EPCs into the tumor. Angiogenesis plays an important role in the development of MM [28]. The interaction between MM cells and the bone marrow microenvironment stimulates the proliferation and migration of endothelial cells. VEGF contributes to the formation of new blood vessels by actively recruiting circulating EPCs. The production of proangiogenic and antiangiogenic factors is also dysregulated in MM. In a previous study, we showed that the promoter region of the PF4 gene, whose product has potent angiostatic effects, is frequently hypermethylated [20], which could tip the balance between the pro- and antiangiogenic factors in favor of aberrant angiogenesis in MM. We have shown here that the overexpression of PF4 or p17-70 in MM cells attenuated the production of VEGF from these cells *in vitro*. We also found significantly reduced serum levels of VEGF in the SCID-rab model of myeloma expressing PF4 or the p17-70 fragment. Other investigators have shown that PF4 inhibits the function of VEGF by disrupting the binding of VEGF to its receptor [18] and disrupting the VEGF-induced intracellular signaling cascade [19]. Our findings suggest that PF4 or the p17-70 fragment also suppresses VEGF function by attenuating the production of this potent proangiogenic factor. However, the exact mechanism by which PF4 exerts its effect on VEGF levels remains to be identified.

The recruitment of circulating EPCs to a tumor site, which leads to aberrant angiogenesis in the tumor, contributes to the tumor's malignant growth and metastasis. PF4 inhibits the functions of two potent proangiogenic factors, FGF and VEGF, that actively recruit circulating endothelial cells to form new blood vessels in the tumor [18,19]. We found that the attenuated production of VEGF in myeloma cells overexpressing PF4 or p17-70 was associated with the reduced migration of EPCs *in vitro*. Our analysis of MVD in the SCID-rab model of myeloma further showed that myelomas overexpressing PF4 or p17-70 exhibited markedly reduced angiogenesis. These findings demonstrate that PF4 exerts its angiostatic effects both *in vitro *and *in vivo*. PF4 suppresses tumor angiogenesis by attenuating VEGF production and causing the impaired migration of EPCs to the tumor site.

Tumor growth depends on angiogenesis. The viral-vector-mediated transfection of the PF4 gene suppressed endothelial cell proliferation and angiogenesis in a mouse xenograft model with glioma, and this was associated with the prolonged survival of the tumor-bearing mice [29]. The tumor-growth-inhibitory effect of PF4 has also been observed in an animal xenograft model of lung cancer [30], and in glioblastoma in an orthotopic human glioblastoma model [31]. No previous report of the effect of PF4 on MM has been available. We established a SCID-rab model of myeloma [27] by inoculating human myeloma cells directly into rabbit tibiae already implanted subcutaneously in SCID mice. We showed that the transfection of the PF4 gene or a sequence encoding p17-70 suppressed the growth of the human myeloma cells in the SCID mice, causing a marked reduction in the tumor volume. Kaplan-Meier analysis further demonstrated that PF4 and p17-70 significantly extended the OS of SICD mice bearing human myeloma xenografts.

PF4 is one of the first cytokines discovered to have antiangiogenic activity in *ex vivo *systems, and its peptide also displays strong antiangiogenic activity [12]. PF4 exerts pleiotropic biological effects, and our study and studies by other investigators strongly indicate that its tumor-growth-inhibitory effect is closely associated with its antiangiogenic activity. MM remains a devastating disease with a dismal outcome, despite improved therapies with novel therapeutic agents. Effectively targeting angiogenesis in MM offers a promising approach to combat the disease. Further studies of PF4 and p17-70 are required to explore the therapeutic potential of this angiostatic agent.

## Conclusions

Our findings indicate that PF4 or p17-70 could be valuable in combating multiple myeloma by disrupting tumor angiogenesis.

## Abbreviations

MM: multiple myeloma; EPCs: endothelial progenitor cells; VEGF: Vascular endothelial growth factor; PF4: Platelet factor 4; FGF2: fibroblast growth factor 2; FBS: fetal bovine serum; ELISA: immunosorbent assay; SDS-PAGE: sodium dodecyl sulfate-polyacrylamide gel electrophoresis; rh-VEGF: human vascular endothelial growth factor; NBCS: New-Born Calf Serum; rh-b-FGF: human basic fibroblast growth factor; MTT: colorimetric 3-(4, 5-dimethylthiazol-2-yl)-2,5 -diphenyltetrazollium bromide; PBS: phosphate-buffered saline; TUNEL: terminal-deoxynucleotidyl-transferase-mediated biotin-dUTP nick end labeling; OS: overall survival.

## Competing interests

The authors declare that they have no competing interests.

## Authors' contributions

LJY carried out the experiments, statistical analysis and drafted the manuscript. JD participated in the design of the study, interpreted all the data, performed the statistical analysis and wrote the manuscript. JH and JFZ performed the cell culture, cell proliferation and lentiviral transfection assay. JH conceived of the study, and participated in its design and coordination. All authors read and approved the final manuscript.

## Authors' information

All authors are affiliated to the address listed below:

Department of Hematology, Changzheng Hospital, The Second Military Medical University, 415 Fengyang Rd, Shanghai 200003, PR China.

## Pre-publication history

The pre-publication history for this paper can be accessed here:

http://www.biomedcentral.com/1471-2407/11/261/prepub
